# Quality Improvement Methodology Facilitates Adherence to Echocardiogram Protocol Measurements

**DOI:** 10.1097/pq9.0000000000000509

**Published:** 2022-01-21

**Authors:** Saira Siddiqui, Eunice Hahn, Garick D. Hill, James Brown, Katherine Lehmkuhl, Christopher Statile

**Affiliations:** * Division of Pediatric Cardiology, Heart Institute, Cincinnati Children’s Hospital Medical Center, Cincinnati, OH; † Department of Pediatrics, University of Cincinnati Medical Center, Cincinnati, Ohio.

## Abstract

**Introduction:**

Local institutional echocardiogram protocols reflect standard measurements as per national guidelines, but adherence to measurements was inconsistent. This inconsistency led to variability in reporting and impacted the use of serial measurements for clinical decision-making. Therefore, we aimed to improve complete adherence to universal and protocol-specific measures for echocardiograms performed for first-time or cardiomyopathy studies from 60% to 90% from July 2019 to February 2020.

**Methods:**

We included all sonographer-performed echocardiograms for first-time or cardiomyopathy protocol studies. We reviewed universal measures and protocol-specific measures for all included studies. We created a scoring system reflecting measurement completion. We used a control chart to measure compliance and established a baseline over 2 months. PDSA cycles over 5 months included interventions such as sonographer education, technical improvements to the measurement toolbar, and group and individual performance feedback.

**Results:**

We reviewed over 4000 studies—the reporting of complete universal measures improved significantly from a median score of 60% to 93%. Protocol-specific measures for first-time studies also showed significant improvement from 62% to 90% adherence. Cardiomyopathy-specific measures demonstrated 87% adherence at baseline, which improved to 95% but then returned to baseline. Sonographer education and toolbar adjustment prompted special cause variation with further improvement following performance feedback. The universal and first-time protocol measures reached 90% adherence with sustained improvement for over 9 months.

**Conclusions:**

We employed quality improvement methodology to improve complete adherence to echocardiographic protocol measurements, thereby facilitating echocardiographic quality and reporting consistency. We plan to spread these interventions to improve adherence to other protocols.

## Abstract 2

Supplemental Digital Content is available in the text.

## INTRODUCTION

The performance of high-quality echocardiograms is vital to accurate diagnosis and medical management. Although qualitative assessment is often used in echocardiogram interpretation, it is wrought with inconsistencies and may adversely impact clinical decision-making.^1,2^ Accurate assessment of left ventricular systolic and diastolic function informs initiation and adjustment of medications in patients with cardiomyopathy.^3^ Suspected changes in left ventricular function may also prompt additional testing, such as cardiac magnetic resonance imaging, which can be cumbersome and challenging, especially for pediatric patients who require sedation.^4^

Quality improvement in echocardiography is a burgeoning field with reports of system and patient-level improvements after implementing this methodology.^5–7^ To attain consistent quality imaging, the American Society of Echocardiography recommends performing several routine measurements in screening echocardiograms and follow-up echocardiograms for structural heart disease.^8^ Also, lesion-specific protocols are recommended to aid in serial assessments.^9–11^ Based on these guidelines, our institution has established protocols for various cardiac lesions and for patients undergoing first-time echocardiograms. However, familiarity with these protocols and adherence to the proposed measures has been inconsistent. To improve the objectivity and consistency of echocardiographic interpretation, we sought to improve adherence to our protocol measurements. The SMART (specific, measurable, actionable, realistic, and timely) aim of our project was to increase complete adherence to universal and protocol-specific measures for patients undergoing echocardiograms for first-time studies or cardiomyopathy from 60% to 90% during July 2019 to February 2020, or 7 months.

## METHODS

### Context

At our institution, 16 registered pediatric cardiac sonographers perform echocardiograms. The echocardiography laboratory performs over 15,000 echocardiograms per year. Pediatric cardiac imaging faculty members review and interpret each echocardiogram. Protocols are distributed to sonographers when they first join our division and again with the start of the academic year. There are universal measures that should be obtained for all echocardiograms, as well as additional protocol-specific measures. Sonographers obtain all necessary images to perform required measurements. The measurements are made on the images after the echocardiogram is completed to reduce the time needed to obtain the echocardiogram. We use Phillips IE33, Phillips Epiq 7, or Siemens SC2000 echocardiogram machines to obtain echocardiographic images and Syngo Dynamics software (Siemens Healthcare, USA) to interpret images. Measurements are performed in Syngo using a toolbar with a drop-down menu listing each of the protocols. Measurements with the toolbar automatically populate the measurement into the report. In addition to the measurements, each report must include image quality and limitations encountered during the scan (body habitus, challenging acoustic windows, lines/devices, lack of patient cooperation, etc). We only reviewed echocardiograms obtained by sonographers and included them in this improvement project to help with consistency. Fellow-obtained echocardiograms were excluded as there is more variation in their level of training.

### Planning the Intervention

**Fig. 1.**
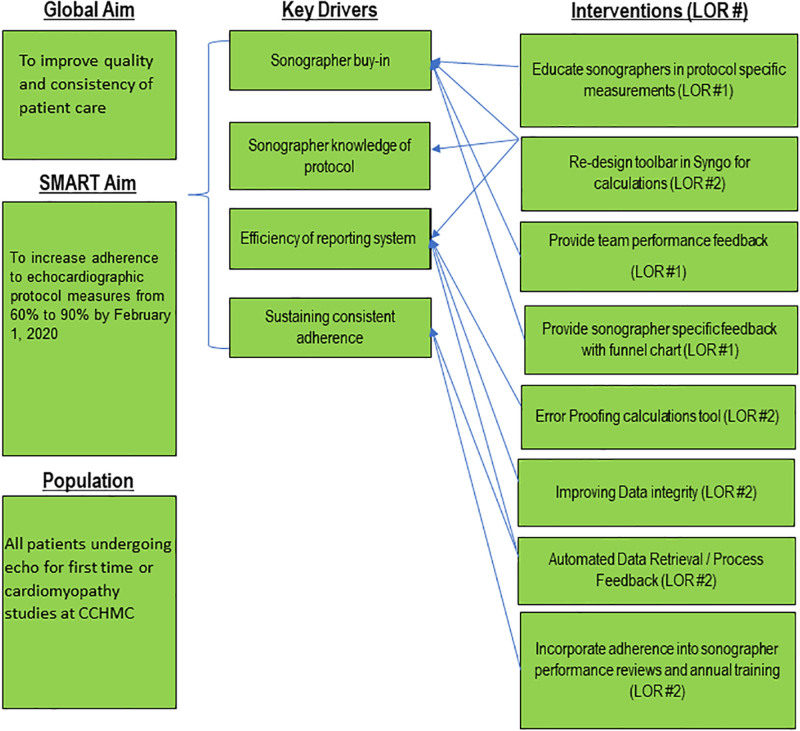
Key driver diagram. The key driver diagram demonstrates the SMART (specific, measurable, achievable, reliable, and timely) aim in the first column. The second column describes the key drivers of change, and the third column includes the interventions made with the level of reliability listed. *LOR*, level of reliability.

We compiled a multidisciplinary team, including a sonographer, echocardiography physician, and quality improvement specialist. We developed a key driver diagram based on feedback from our sonographer and physician team (Fig. 1). Given the high volume of echocardiograms performed at our center, 2 months provided sufficient echocardiograms to establish a baseline for all measurements. We included all sonographer-performed echocardiograms for first-time or cardiomyopathy protocol studies. These protocols were selected because they are the most frequently utilized within our institution, and their measurements have been well validated.^12,13^ We measured adherence to both universal measures and protocol-specific measures across all studies.

### Development of Aim and Outcome Measures

Our primary outcome measure was percent adherence to universal measures, which were performed on all studies. Syngo Dynamics software (Siemens Healthcare, USA) was used to identify echocardiograms by protocol and to extract all relevant measures. After extracting the data, we converted the measures into a scoring system reflecting measurement completion. Based on the American Society of Echocardiography guidelines, we established necessary universal and protocol-specific measures and awarded one point for each completed measurement (**See table, Supplemental Digital Content 1,** which lists the universal and protocol-specific measures. One point was given for each completed measurement, with the total attainable points listed. The LV area d, LV area s, LV major d, and LV major s are four measurements used to calculate the left ventricular ejection fraction by the 5/6 area length method, which was counted as one point. http://links.lww.com/PQ9/A345).^13^ A total of 10 points were attainable for universal measures. In addition, first-time study measures could attain an additional seven protocol-specific points, and cardiomyopathy protocol measures could attain an additional nine protocol-specific points.

We established a baseline of measurement adherence by reviewing echocardiograms performed under the first-time study or cardiomyopathy protocols for 2 months. This baseline included 550 echocardiograms with an overall 60% adherence to universal measures. For first-time protocol studies, there was adherence of 62% to first-time-specific study measures. For cardiomyopathy protocol studies, there was 87% adherence to cardiomyopathy-specific measures. Given the unexpectedly high baseline adherence to cardiomyopathy measures, we decided to increase our goal for that specific protocol to 95%. At the beginning of the project, we presented the project aims and baseline data to all physicians and sonographers at a monthly echocardiography laboratory meeting. In addition, we surveyed the sonographers regarding their experience with the interventions and their impact on sonographer efficiency as a balancing measure.

### Improvement Activities

We performed four plan-do-study-act (PDSA) cycles to implement our SMART aim. We designed all interventions after discussion among the echocardiography physician, sonographer, and quality improvement team members. We focused our interventions on the key drivers of sonographer buy-in, sonographer knowledge of the protocols, the efficiency of the reporting process, and sustaining consistent adherence. Our PDSA cycles included (1) sonographer education through conferences and group review of the individual protocols, (2) technical improvements to the measurements toolbar, (3) group performance feedback, and (4) individual performance feedback.

### Sonographer Education

Our first intervention consisted of educational sessions to improve sonographers’ knowledge of the protocols and awareness of each protocol’s measurements. These sessions included presentations reviewing the first-time study and cardiomyopathy protocols and the American Society of Echocardiography guidelines on performing measurements accurately.^8^ During these sessions, we uncovered a knowledge deficit among many sonographers about the standard measurements. Also, many reported being unaware of all the required universal measurements. These findings prompted our subsequent intervention aimed at this knowledge gap.

### Technical Improvements

**Fig. 2.**
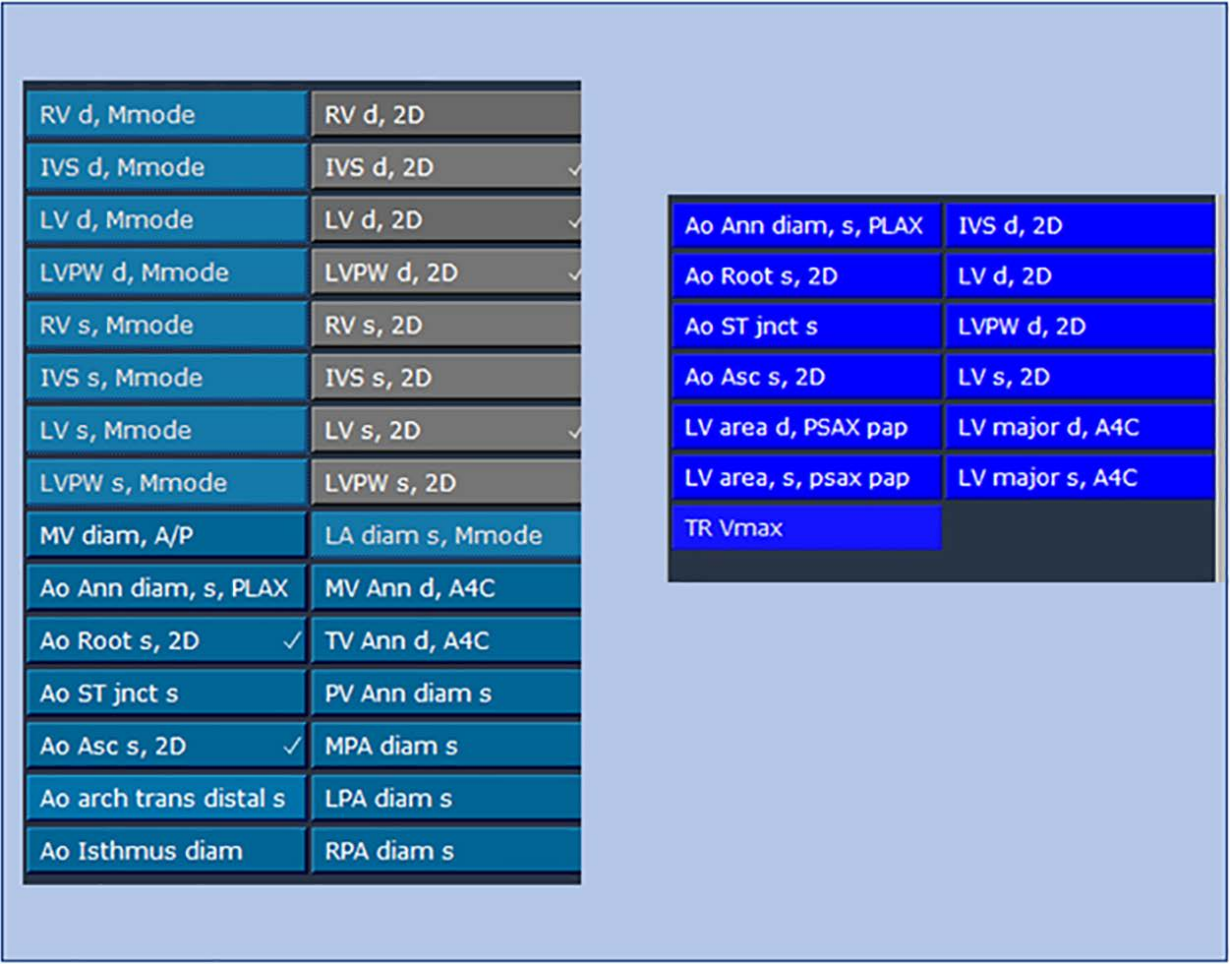
Upgraded toolbar for universal measures. The left-sided panel shows the previous toolbar for universal measures with multiple different colors and several unnecessary measurements. The right-sided panel shows the upgraded toolbar with only the necessary measurements included. This intervention prompted special cause variation in our adherence to universal measures.

For our next intervention, we addressed the technical process of performing the measurements. With the assistance of the information technology support team, we changed the toolbar. Previously the toolbar had been redundant with confusing titles for the various protocols and measurements (Fig. 2). Also, the protocol tabs did not completely encompass all the necessary measures for each protocol. For example, both a “universal measures” tab and a “universal measurements” tab were listed, but neither of these tabs included all the necessary universal measures. This format was particularly challenging for newer sonographers who had to learn where each measurement was listed in the toolbar. Therefore, a tab was created for each protocol in the updated toolbar and included all the necessary protocol-specific measurements. We also included a separate universal measures tab. We waited until 2 weeks after implementing the update to remove the previous protocol tabs to allow familiarity with the updated toolbar to develop. By removing the previous protocol tabs, we ensured that only our updated toolbar was available for use.

### Performance Feedback

Our final interventions included performance feedback. The efficacy of performance feedback at both the group and individual levels has been previously demonstrated.^14^ Group feedback was provided by posting statistical process control charts in the sonographer team room and discussing them in the monthly echocardiography laboratory meeting. Individual feedback, including funnel charts highlighting the individual sonographer’s performance relative to the group, was provided in a sealed envelope. Verbal feedback was provided during semiannual performance reviews and informally throughout the year if trends were noted.

### Studying the Interventions

#### Analysis

We analyzed our data using a statistical process control chart. We used a P chart to plot data over time with upper and lower control limits representing the system’s inherent variation. We identified special cause variation as an external change to the system. Three rules defined special cause variation: a single point outside the control limits (*P* = 0.0015), six consecutive points trending up or down (*P* = 0.00278), or eight or more consecutive points above or below the centerline (*P* = 0.0078).^15^

We performed an anonymous retrospective sonographer survey as a balancing measure. The survey queried whether updating the toolbar impacted sonographer efficiency and ease of measurement completion. This survey accounted for both sonographers who performed echocardiograms before the toolbar update and experienced the change in practice and those who joined our institution after implementing the updated toolbar.

#### Ethical Considerations

As per the CCHMC Institutional Review Board’s guidance for QI projects, this initiative did not constitute human subjects research and was exempt from IRB approval. Despite the involvement of human subjects in a QI project rather than a clinical research study, informed consent documentation was not required. The physicians and sonographers involved in this project were aware that their participation was voluntary. We used the SQUIRE (Standards for Quality Improvement Reporting Excellence) 2.0 guidelines to report our findings.^16^

## RESULTS

We reviewed and included 4023 studies for analysis. Of these studies, 2113 (53%) were first-time studies, and the remaining 1910 (47%) were cardiomyopathy studies. Sixteen cardiac sonographers performed echocardiograms included in our study.

### Universal Measures

**Fig. 3.**
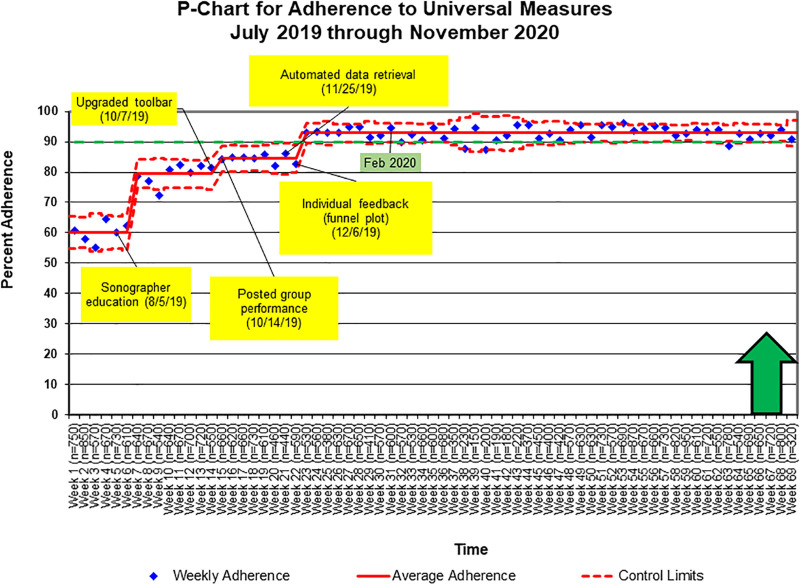
Control chart demonstrating special cause variation in adherence to universal measures with improvement from 60% to 93%. The yellow boxes indicate interventions. The green box indicates the established goal date for improved adherence. The green arrow indicates the direction of anticipated change. The n value is the total number of attainable points for each week.

Over 7 months, reporting of complete universal measures improved significantly from a median score of 60% to 93% (Fig. 3). Sonographers sustained this improvement for over 9 months. Sonographer education and adjustment to the toolbar prompted special cause variation with further improvement following individual performance feedback.

**Fig. 4.**
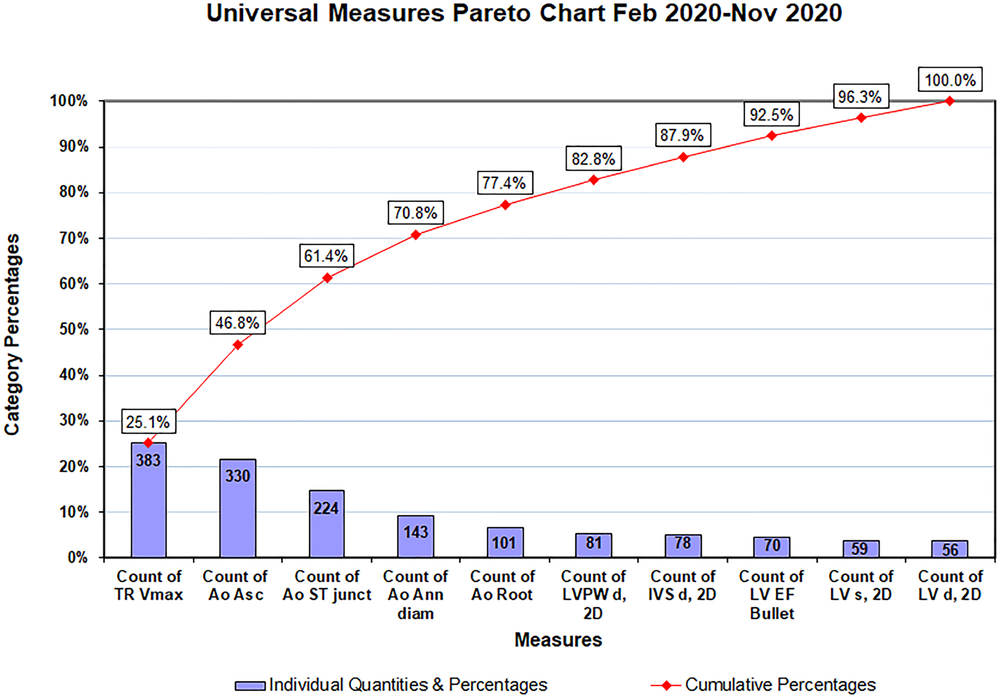
Pareto Chart presenting the most common of the universal measures missed between February and November 2020.

After achieving our goal, there were 330 failures (between weeks 23 and 69), with less than 90% of the universal measures reported. Of these failures, 153 (46%) had technically limited images due to patient-related factors. Of the remaining 177 failures, 31 (18%) occurred before our goal improvement date of February 1, 2020. The average score among the 146 failures that occurred after the goal improvement date was 6.7/10. A Pareto chart identified the most common missing measurements, which we then emphasized in subsequent educational sessions (Fig. 4). Also, we found that 17 of the 146 failures (12%) that occurred after the goal improvement date resulted from one technician. This technician started in June 2020 during the coronavirus pandemic, which may have impacted her ability to get one-on-one teaching from the other technicians and direct feedback from physicians due to physical separation between the sonographers and physicians.

### Protocol-specific Measures

**Fig. 5.**
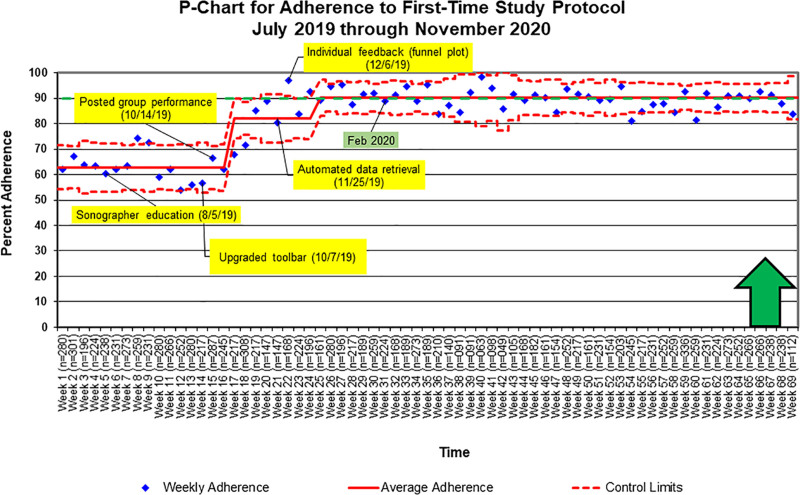
Control chart demonstrating special-cause variation in first-time study protocol measures with improvement from 62% to 90%. The yellow boxes indicate interventions. The green box indicates the established goal date for improved adherence. The green arrow indicates the direction of anticipated change. The n value is the total number of attainable points for each week.

Protocol-specific measures for first-time studies significantly improved from 62% to 90% adherence (Fig. 5). Also, there was consistent adherence at goal after week 25 (12/23/2019) with only 2 points below the lower control limit.

**Fig. 6.**
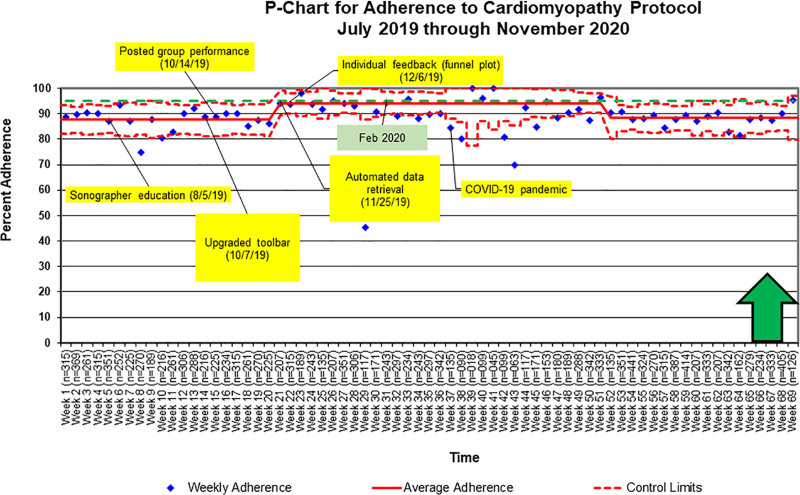
Control chart demonstrating initial special cause variation for cardiomyopathy protocol measures from a baseline of 87% to 95% adherence, which subsequently returned to baseline. The yellow boxes indicate interventions. The green box indicates the established goal date for improved adherence. The green arrow indicates the direction of anticipated change. The n value is the total number of attainable points for each week.

Cardiomyopathy-specific measures demonstrated 87% adherence at baseline, which mildly improved to 95% and then returned to an average adherence of 88% (Fig. 6). There was a cluster of a few weeks with adherence below the lower control limit (weeks 37, 38, 42, 43, and 45). Upon analyzing these data, we noted some failures due to patient-related factors. We utilize the cardiomyopathy protocol primarily during outpatient visits. Our clinic was running at 50% capacity due to the coronavirus-19 pandemic during this time with limited staffing and the completion of more focused and efficient studies. The impact of these systems-changes likely caused a more significant impact on the overall weekly adherence. Despite these few data points, the overall adherence was at goal.

### Balancing Measures

We obtained responses from all 16 sonographers regarding the utility of the updated toolbar tab and the impact of the changes to sonographer efficiency. Of the 16 sonographers, 11 had completed echocardiograms at our institution before the toolbar tab change in October 2019. Of the 11 sonographers, 9 (82%) and 7 (64%) reported that the new toolbar “never” or “rarely” required more time to complete universal and protocol-specific measures, respectively. All sonographers reported improved awareness of the required measures and ease of locating measures. For the remaining five sonographers who joined our institution after the change in the toolbar tab, none reported that measurement completion impeded workflow.

## DISCUSSION

Our project demonstrates that quality improvement methodology can improve adherence to echocardiographic protocol measurements within a short period. These improvements can facilitate the consistency of echocardiographic quality and reporting. We focused on measurement adherence as the first step toward improving the quality and consistency of our measures with > 90% adherence and sustained improvement demonstrated across all included measures. Our team exceeded our improvement goal despite the inherent patient-level limitations of echocardiography and variability in sonographer educational background and schedules. Importantly, we implemented these changes with our cardiac sonographers’ support, who expressed largely positive feedback for the changes. While not our primary outcome, improving imaging quality and measurement consistency may also improve clinical outcomes. We use serial echocardiographic measurements in patients with cardiac disease to guide clinical decision-making, including initiation of medications and timing of surgical referral.^17–19^

This study is unique in pediatric echocardiography as it employed quality improvement science to improve measurement completion. Prior projects have focused on the quality of echocardiographic measurements or correlation with other invasive cardiac parameters rather than on measurement adherence.^20–23^ We identified incomplete measurement adherence and missing measurements, rather than inaccurate information not identified before report completion. We then designed our study to address sonographer- and systems-level challenges. The key to our success was sonographer buy-in. We consistently sought out sonographer feedback about improvement activities in both formal and informal settings. Personal outreach to sonographers greatly improved communication and commitment from the team. Senior sonographers were empowered to share their experiences and assist in training the newer sonographers. Also, we reinforced educational components individually and in group settings to reach all sonographers.

Many of our interventions can be generalized. For example, when made in conjunction with cardiac sonographers, improvements to the reporting system can be very helpful in obtaining sonographer buy-in while also improving efficiency. For our reporting system, the toolbar upgrade was instrumental in streamlining the reporting process. In addition, interventions such as sonographer education and feedback are technically facile to incorporate into the echocardiography laboratory practice.

There are limitations to the generalizability of our study. Quality improvement methodology and projects are well-incorporated into our institutional framework. Other centers may find that team buy-in and awareness of quality improvement methodologies may be challenging. Also, other centers may lack personnel specifically trained in pediatric cardiac studies, necessitating more intensive sonographer education regarding measurements. Finally, the technical aspects of adjusting echocardiographic reporting may be more difficult in some centers. We are fortunate to have a robust technical team that assists in software issues and automated data retrieval to ensure inclusion and analysis of all encounters.

There are other limitations to our project. We did not account for the patient experience. Because the images obtained are standard for each protocol and we obtain the measurements after completing the echocardiogram, we did not expect significant changes in the echocardiogram duration or increased clinical delays. Also, we had some turnover in our sonographer cohort during the study. Therefore, the efficacy of some of our earlier interventions may not apply to our current cohort. While our imaging faculty knew the quality improvement project and were expected to ensure the report’s measurement completion, we did not specifically target PDSA cycles toward them. Their involvement could be an opportunity for sustainable improvement in the future. Finally, this was a single-center study. Other institutions have not validated our scoring system.

Sustainability and spread are vital albeit challenging aspects of quality improvement projects. As a result of our project, changes to new sonographer training and automated reports have been incorporated into our workflow to help sustain our improvement. Also, we are working with imaging faculty to include adherence to measurements as part of the sonographer review process. We intend to spread our quality improvement interventions to improve adherence to other protocol-specific measures, including those for congenital heart disease. Improved measurement adherence is essential in improving the quality of cardiac imaging and reporting used for clinical decision-making. Many echocardiographic measurements included in our protocol automatically generate validated z-scores standardized to patient body surface area.^24,25^ Consistent and accurate measurements can facilitate early detection of cardiac abnormalities and impact patient care.

## CONCLUSIONS

Utilizing a team-based approach, we incorporated interventions targeting sonographer education, performance feedback, and reporting systems to improve adherence to our echocardiographic universal and protocol measures to greater than 90% with sustained improvement over 9 months. These interventions can be applied to other protocols and generalized to other centers for improvement in echocardiographic reporting.

## DISCLOSURE

The authors have no financial interest to declare in relation to the content of this article.

## ACKNOWLEDGEMENTS

We thank the cardiac sonographers who participated in this study and acknowledge Todd Dupuis and Nichole Allen for their assistance with the technical aspects.

## Supplementary Material


