# Supplementary material for: Quality Improvement Methodology Facilitates Adherence to Echocardiogram Protocol Measurements
Source: Pediatr Qual Saf. 2022 Jan 21;7(1):e509. doi: 10.1097/pq9.0000000000000509 (PMC8782111; doi:10.1097/pq9.0000000000000509)
Supplement: Supplementary file 1 [file pqs-7-e509-s001.pdf]

Protocol	Universal Measures	First-time Study	Cardiomyopathy
Measures (1 point each)	Aortic annulus	Mitral valve annulus	Mitral valve E velocity
	Aortic root	Tricuspid valve annulus	Mitral valve A velocity
	Sinotubular junction	Pulmonary valve annulus	Mitral valve deceleration time
	Ascending aorta	Right pulmonary artery	Mitral valve lateral wall tissue Doppler e’
	Interventricular septum, diastole (M-mode)	Left pulmonary artery	Mitral valve lateral wall tissue Doppler a’
	Left ventricle, diastole (M-mode)	Aortic isthmus	Mitral valve medial wall tissue Doppler e’
	Left ventricle, systole (M-mode)	Distal transverse arch	Mitral valve medial wall tissue Doppler a’
	Left ventricular posterior wall dimension, diastole (M-mode)		Tissue annular plane systolic excursion (TAPSE)
	Left ventricular ejection fraction		Left atrial volume
	Tricuspid regurgitant velocity OR Insufficient tricuspid regurgitation for right ventricular systolic pressure estimate		
Total points	10	7	9
